# Social inequalities in mild and severe myocardial infarction: how large is the gap in health expectancies?

**DOI:** 10.1186/s12889-021-10236-7

**Published:** 2021-02-01

**Authors:** Juliane Tetzlaff, Siegfried Geyer, Mechthild Westhoff-Bleck, Stefanie Sperlich, Jelena Epping, Fabian Tetzlaff

**Affiliations:** 1 Medical Sociology Unit, Hannover Medical School, Hanover, Germany; 2 Department of Cardiology and Angiology, Hannover Medical School, Hanover, Germany; 3 Institute for General Practice, Hannover Medical School, Hanover, Germany

**Keywords:** Myocardial infarction, Social inequalities, Incidence, Mortality, Health expectancies, Germany

## Abstract

**Background:**

Acute myocardial infarction (MI) remains a frequent health event and a major contributor to long-term impairments globally. So far, research on social inequalities in MI incidence and mortality with respect to MI severity is limited. Furthermore, evidence is lacking on disparities in the length of life affected by MI. This study investigates social inequalities in MI incidence and mortality as well as in life years free of MI and affected by the consequences of mild or severe MI.

**Methods:**

The study is based on data of a large German statutory health insurance provider covering the years 2008 to 2017 (*N* = 1,253,083). Income inequalities in MI incidence and mortality risks and in life years with mild or severe MI and without MI were analysed using multistate analyses. The assessment of MI severity is based on diagnosed heart failure causing physical limitations.

**Results:**

During the study period a total of 39,832 mild MI, 22,844 severe MI, 276,582 deaths without MI, 15,120 deaths after mild MI and 16,495 deaths after severe MI occurred. Clear inequalities were found in MI incidence and mortality, which were strongest among men and in severe MI incidence. Moreover, substantial inequalities were found in life years free of MI in both genders to the disadvantage of those with low incomes and increased life years after mild MI in men with higher incomes. Life years after severe MI were similar across income groups.

**Conclusions:**

Social inequalities in MI incidence and mortality risks led to clear disparities in the length of life free of MI with men with low incomes being most disadvantaged. Our findings stress the importance of primary and secondary prevention focusing especially on socially disadvantaged groups.

**Supplementary Information:**

The online version contains supplementary material available at 10.1186/s12889-021-10236-7.

## Background

Acute myocardial infarction (MI) is one of the major causes of hospital admissions leading often to long-term impairments and premature death in the affected population [1–4]. MI often triggers a sudden deterioration in cardiovascular health resulting in increased risks of further cardiovascular acute events like recurrent MIs or stroke, poor quality of life and impaired functional health [5–11]. Although considerable progress has been made in treatment and prevention, population ageing and declining mortality after MI are assumed to increase the burden of MI globally [1, 12]. There is a large body of research that confirms the close link between SES and MI incidence and mortality risks [13–22]. However, so far research investigating social inequalities in MI incidence and mortality with respect to the severity of MI is still insufficient. In particular, little is known about how socioeconomic status (SES) affects the average lifetime spent free of MI and after mild or severe MI.

Major factors contributing to social inequalities in MI incidence and mortality risks lie in the increased prevalence of cardiovascular risk factors and harmful health behaviours in lower SES groups [20, 23, 24]. In the German population these inequalities were found in both, the prevalence of risk factors (e.g. hypertension, hyperlipidaemia, high Body Mass Index, and diabetes) [25–27] and risky health behaviour (e.g. smoking, physical inactivity) [28–30] which contribute to the substantial inequalities in MI incidence and mortality risks [31–34]. The prevalence of cardiovascular risk factors and harmful health-related behaviour also differs significantly between men and women. This applies in particular to smoking, unhealthy nutrition, hazardous alcohol consumption and high blood pressure, which are more prevalent in men than in women [35–37], while no clear gender differences were found for exercise and in the prevalence of dyslipidaemia and diabetes mellitus [36, 38, 39]. Moreover, some studies suggest that social inequalities in cardiovascular risk factors and harmful health-related behaviour might be stronger in women than in men, e.g. in smoking and diabetes mellitus [24, 36]. Studies also indicate that MIs are often severe in individuals with low SES and cause high mortality [17, 40, 41], increased risks of hospital readmission and of substantial and permanent deterioration in cardiovascular health (especially chronic heart failure, recurrent MI) [19, 21, 42–44]. However, so far little is known on the gap in average lifetime affected by the consequences of MI between SES groups and how inequalities in life years differ with respect to MI severity.

The aim of the study is to shed light on the interplay between MI incidence and mortality in different SES groups and on the resulting disparities in health expectancies among the German population, i.e. the number of life years free of MI and that affected by mild or severe MI. In addition, gender differences in health expectancies and the related social inequalities are studied. This retrospective cohort study is based on health insurance claims data and investigates social inequalities in MI incidence and mortality with respect to MI severity among the elderly population based on the data of a large German health insurance provider. In order to investigate the length of life free of MI and affected by MI at population level, it is crucial to analyse this interplay between MI incidence and mortality without and after MI: While higher MI incidence rates may increase the expected number of life years after MI in individuals with low SES, increased mortality risks may counterbalance this tendency by shortening the length of survival after MI. In contrast, individuals with high SES may have lower incidence rates, but also lower mortality after MI. Since the extent of the impairments in daily life depends on MI severity, this study focuses in particular on inequalities in MI severity and in the length of life affected by mild or severe MI.

In more detail, the study addresses the following research questions:

1. Are there SES inequalities in MI incidence and mortality without and after MI? Do these inequalities differ in terms of MI severity and between genders?
2. Are there SES inequalities in the number of life years free of MI and in life years after MI? Do these inequalities differ with respect to mild and severe MI and between genders?

## Methods

### Data

The analyses are based on claims data of a large statutory German health insurer, the AOK Niedersachsen (AOKN), i.e. on data routinely collected for accounting purposes including the entire insurance population. The AOKN covers about a third of the total population of the federal state Lower Saxony [45]. As part of the welfare-state system, being insured within the statutory health insurance system is mandatory for all German inhabitants below a certain income threshold. Above this threshold, individuals can chose between statutory and private health insurance. Approximately 90% of all German inhabitants are insured within the statutory health insurance system [46]. Statutory health insurance data depict health care activities fairly complete as all medical services causing payments to the providers are recorded. Therefore, the data contain detailed information on diagnoses and medical procedures which can be used for statistical analyses. The study includes the complete elderly population insured with the AOKN within the study period 2008–2017 regardless of their health status or frequency of healthcare service use. Since the number of MIs and deaths is low at younger ages, the age range included was restricted to 50 years and older. Comparative analyses have shown that the data are representative for the total German population in terms of age and sex distributions but differ with respect to socioeconomic characteristics as individuals with higher incomes and higher occupational qualifications are underrepresented [47]. This may result in higher crude morbidity rates than in the total German population, if no adjustment for these differences in SES characteristics is made.

### Definition MI incidence and death events

*Incident cases* of MI were identified based on inpatient ICD-10 diagnoses I21.0 to I21.9. In order to distinguish between recurrent and first MIs, lookback periods of 1 year were applied that had to be free of a MI diagnosis in incident cases. Incident cases were classified as “*severe*”, if a person either 1) had a diagnosis of chronic heart failure during the hospital stay initiated by the MI with New York Heart Association (NYHA) classification 3 or 4 (ICD-10 I50.04-I50.05 or I50.13-I50.14), which indicate marked or severe physical limitations at moderate or low activity level [48], or 2) died within 7 days after MI.MI cases of patients surviving longer than 7 days without or with mild heart failure symptoms (NYHA 1 or 2, no or slight limitations during ordinary activity [48]) were classified as “*mild*” MI. Furthermore, the data contain information on the exact date of death for all individuals deceased within the study period. Using this information, three different events can be distinguished: death without MI, death after mild MI and death after severe MI.

### Income

Social inequalities in MI incidence and mortality were analysed using income information of the insured individuals. Income represents a major SES indicator since it determines the financial resources available to individuals and thus has a decisive influence on their chances of achieving health-promoting living conditions and behaviours [49]. Since insurance premiums depend on income, the data contain detailed information on individual income from salaries and pension payments received from the German Pension Fund. For this study, individuals were assigned to three groups according to their income level relative to the average income from salaries in the total West German population: ≤60% (low), > 60% to ≤80% (middle), and > 80% (higher). Official statistics provide information on the average income from salaries for each year of the study period. The study population was assigned to income groups based on the average income of the respective year of observation. In this way, the grouping may vary between years in absolute terms but remains constant in relative terms, accounting for rising income levels over time. Individuals with missing information on income were excluded. Within the study period, the proportion of insured individuals with missing information on income amounted to 8% (6% men, 10% women).

### Statistical methods

**Fig. 1:**
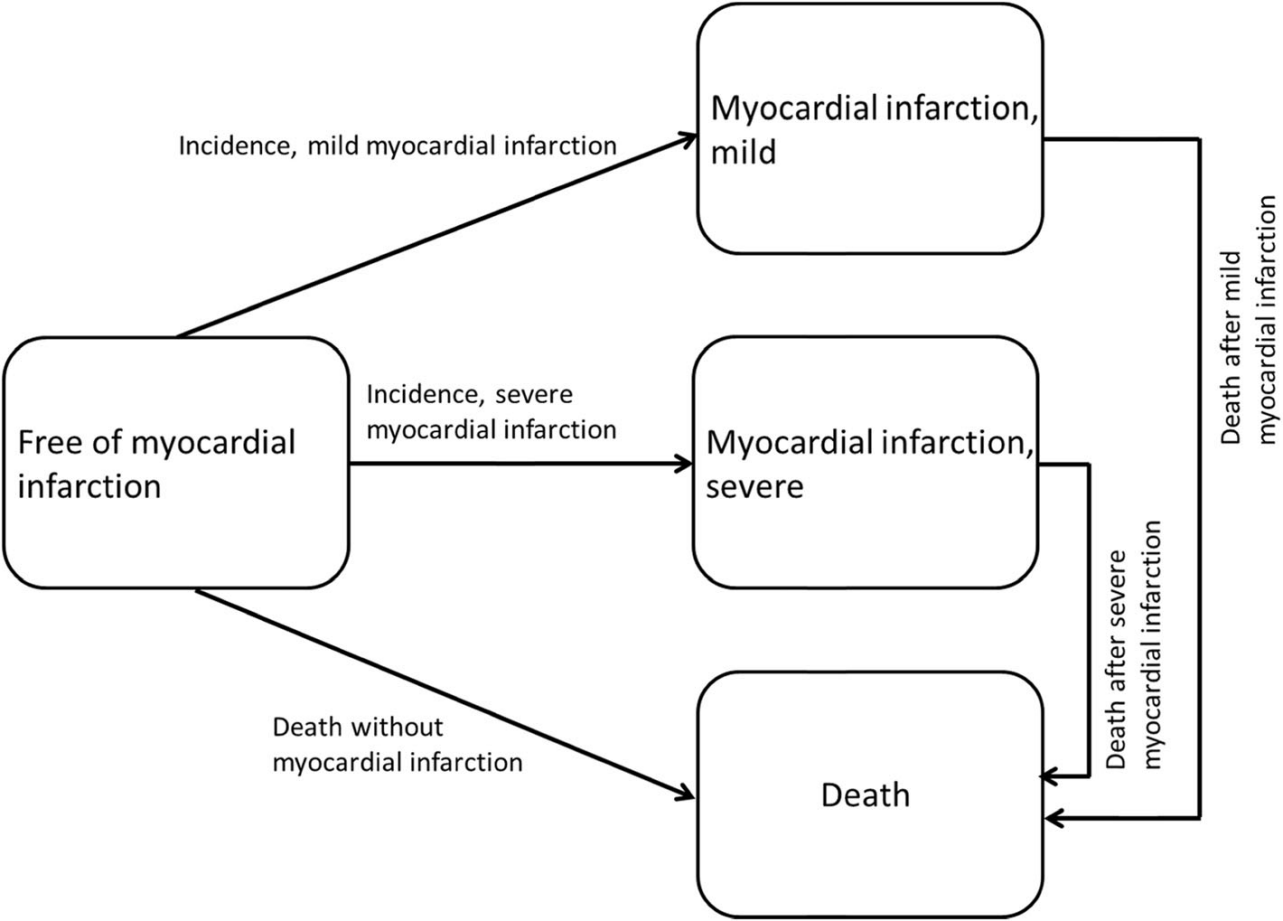
Illness-death-model defining two transitions to myocardial infarction incidence and three transitions to death

Income inequalities in the expected number of life years spent free of MI, after a mild MI, and after a severe MI were analysed using multistate survival models. To account for the complex interplay between incidence and mortality, age-specific rates of MI incidence and mortality with and without MI are needed. These estimates are based on a four-state illness-death model defining two transitions to incidence and three transitions to death: 1) MI incidence, low severity (free of MI to mild MI), 2) MI incidence, high severity (free of MI to severe MI), 3) death without myocardial infarction (free of MI to death), 4) death after MI, low severity (mild MI to death), 5) death after MI, high severity (severe MI to death) (Fig. 1).

**Fig. 2:**
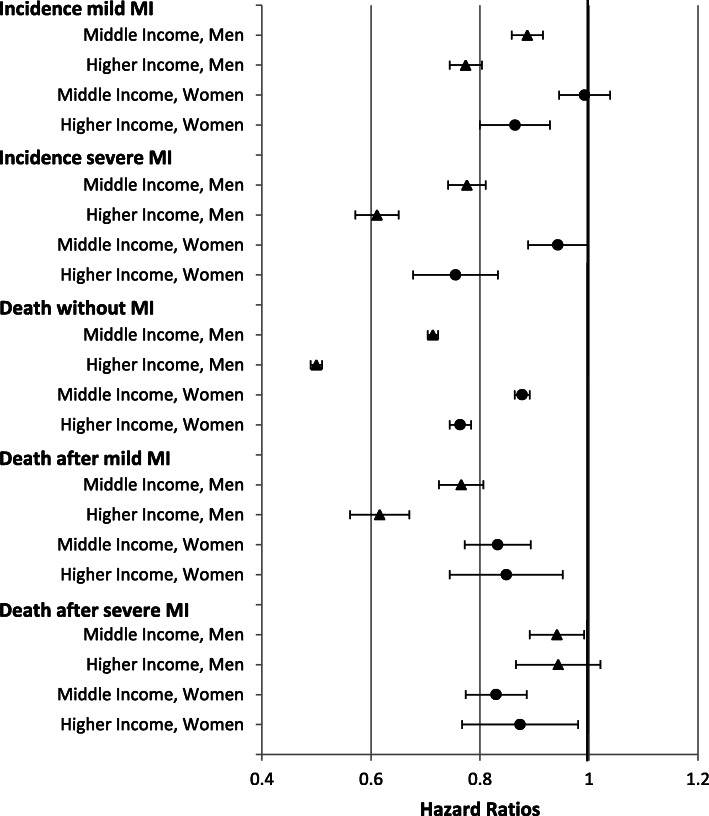
Income inequalities in the risks (HR) of myocardial infarction incidence and death by degree of myocardial infarction severity and gender (reference: low-income group). Note: HR Hazard Ratio; 95%-CI bootstrapped (with replacement) using 1000 replications; all analyses are controlled for age in single-year age groups (as second-degree polynomial)

The survival models were estimated for each type of transition separately using exponential models with constant baseline hazard. All models are stratified by gender and include age as covariate (centred to the middle of the age interval 50 to 95+ and included as second degree polynomial). First, income gradients were estimated for each of the five transitions using the low-income group as reference category (Fig. 2). For an alternative presentation, a figure setting the higher-income group as reference is included in the supporting materials (Additional file 1, Fig. S1). In a second step, incidence and mortality rates were estimated for each income group separately. From these models, age-specific rates of MI incidence and mortality were predicted. These rates were used as input for the multistate life table analyses to estimate health expectancies at age 50, i.e. the expected number of remaining life years free of MI and after mild or severe MI. To assess the model fit, the predicted incidence and mortality rates were plotted against the observed rates. Overall, the predicted rates fit the observed rates well, with the observed rates tending to fluctuate more at old age and for death after MI due to the limited number of cases (Additional file 1, Fig. S2 and S3). Sensitivity analyses were performed with Cox proportional hazard models and different parametric survival models, e.g. Gompertz models, which however did not change the results.

## Results

Between 2008 and 2017, a total of 1,253,083 insured individuals aged 50 years and above were included in the analyses. Among the study population, a total of 62,676 MI incident cases were observed (64% mild MIs), 31,615 deaths after MI (48% after mild MI), and 276,582 deaths without MI (Table 1). Since the age range is restricted to the elderly, a major share of the study population is retired, leading to low proportions of individuals with higher incomes. This applies especially to women. Detailed information on the number of events, exposures in person-years, and the number of individuals by gender, income group and type of transition can be found in Table 1.

**Table 1:** Characteristics of the study population: the number of individuals, exposures in person-years, and number of events by transition, income group and gender

		Men				Women			
		Low	Middle	Higher	Total	Low	Middle	Higher	Total
**Incidence**	*Individuals*	282,457 (49%)	115,988 (20%)	176,322 (31%)	574,767 (100%)	530,246 (78%)	85,682 (13%)	62,388 (9%)	678,316 (100%)
**mild MI**	*Exposures*	2,003,937 (48%)	841,534 (20%)	1,356,445 (32%)	4,201,915 (100%)	4,032,069 (78%)	646,959 (13%)	474,169 (9%)	5,153,197 (100%)
	*Events*	13,436 (58%)	5378 (23%)	4237 (18%)	23,051 (100%)	13,888 (83%)	2087 (12%)	806 (5%)	16,781 (100%)
**Incidence**	*Individuals*	282,457 (49%)	115,988 (20%)	176,322 (31%)	574,767 (100%)	530,246 (78%)	85,682 (13%)	62,388 (9%)	678,316 (100%)
**severe MI**	*Exposures*	2,003,937 (48%)	841,534 (20%)	1,356,445 (32%)	4,201,915 (100%)	4,032,069 (78%)	646,959 (13%)	474,169 (9%)	5,153,197 (100%)
	*Events*	7467 (64%)	2854 (25%)	1284 (11%)	11,605 (100%)	9423 (84%)	1395 (12%)	421 (4%)	11,239 (100%)
**Death without MI**	*Individuals*	282,457 (49%)	115,988 (20%)	176,322 (31%)	574,767 (100%)	530,246 (78%)	85,682 (13%)	62,388 (9%)	678,316 (100%)
	*Exposures*	2,003,974 (48%)	841,546 (20%)	1,356,496 (32%)	4,202,016 (100%)	4,032,101 (78%)	646,966 (13%)	474,173 (9%)	5,153,241 (100%)
	*Events*	80,146 (67%)	29,154 (24%)	10,964 (9%)	120,264 (100%)	131,504 (84%)	18,496 (12%)	6318 (4%)	156,318 (100%)
**Death after**	*Individuals*	13,436 (58%)	5378 (23%)	4237 (18%)	23,051 (100%)	13,888 (83%)	2087 (12%)	806 (5%)	16,781 (100%)
**mild MI**	*Exposures*	50,399 (60%)	19,326 (23%)	13,727 (16%)	83,452 (100%)	47,016 (83%)	7299 (13%)	2646 (5%)	56,962 (100%)
	*Events*	5225 (66%)	2023 (26%)	617 (8%)	7865 (100%)	6057 (83%)	894 (12%)	304 (4%)	7255 (100%)
**Death after**	*Individuals*	7467 (64%)	2854 (25%)	1284 (11%)	11,605 (100%)	9423 (84%)	1395 (12%)	421 (4%)	11,239 (100%)
**severe MI**	*Exposures*	13,027 (66%)	4658 (24%)	1906 (10%)	19,591 (100%)	14,557 (83%)	2346 (13%)	670 (4%)	17,572 (100%)
	*Events*	5296 (66%)	2049 (25%)	711 (9%)	8056 (100%)	7089 (84%)	1046 (12%)	304 (4%)	8439 (100%)

### Income inequalities in MI incidence and mortality risks

The analyses revealed substantial income inequalities in the risk of MI incidence, disadvantaging those with lower incomes. Income gradients were strongest in men and in severe MIs in both genders (Fig. 2). Inequalities were also substantial in the risk of death after mild MI. Weaker, but still evident inequalities were also found for death after severe MI. Overall, income inequalities in incidence and mortality risks tend to be higher in men than in women (Fig. 2).

### Income inequalities in life years free of MI and after MI

**Fig. 3:**
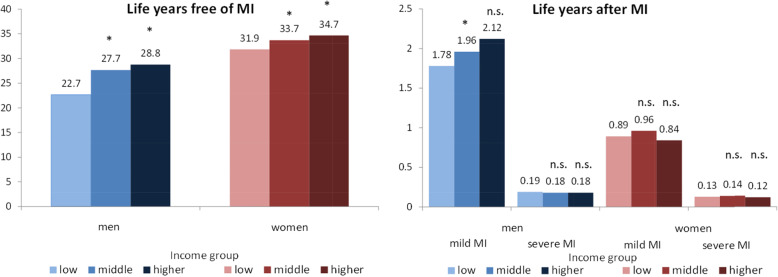
Expected number of life years free of myocardial infarction (MI) and after mild and severe MI at age 50 by gender and income group. Note: Significantly different from the next lower income group based on 95%-Confidence intervals (CI), CI bootstrapped (with replacement) using 1000 replications, MI myocardial infarction

With regard to the expected length of life free of MI, an income gradient emerged, with men and women with higher incomes being most advantaged. These inequalities were particularly strong in men with a difference of 6.1 life years free of MI between the low- and the higher- income group (22.7 years vs. 28.8 years at age 50). Among women, the gap between income groups is smaller but still evident and amounts to 2.8 years (31.9 vs. 34.7 years). Overall, women can expect more life years free of MI, since mortality and incidence rates are considerably lower than among men (Fig. 3).

In men, inequalities in life years after mild MI were also considerable, with life years ranging from 1.78 years in the low- to 2.12 years in the higher-income group, while in women no marked income inequalities were found. Compared to the length of life after mild MI, the number of life years after severe MI is low in both genders and differences between income groups were minor (Fig. 3).

## Discussion

The study shows that differences between income groups in MI incidence and mortality risks are considerable, leading to substantial inequalities in the expected length of life free of MI and after MI. The analyses also reveal that inequalities vary in level according to the severity of MI and gender. Inequalities in MI incidence tend to be stronger in men than in women and are most pronounced in severe MI incidence. Similar patterns were also found for mortality risks without MI and after mild MI, while income inequalities in the risk of death after severe MI were weaker in men than in women. These different patterns of inequalities between men and women are also reflected in the number of life years free of MI and affected by MI. While clear inequalities were found in life years free of MI for both genders, the gap between the low and higher-income group was more than twice as high for men as for women. Men with low incomes are even doubly disadvantaged, as they can expect not only fewer healthy years but also less years after mild MI due to higher mortality risks. In contrast, no significant inequalities in life years after mild and severe MI were found in women and in men after severe MI. While some studies reported a substantial gap in total life expectancy between income and educational groups among the German population [50–52], research on social inequalities in health expectancies is still very limited. This holds especially true for studies focusing on MI and other specific diseases. This may be due to the limited availability of data that provide information on SES, incidence and mortality. In order to analyse how many years of total life expectancy can be attributed to years free of MI and years after disease onset, the interplay in inequalities between MI incidence and mortality with and without MI has been studied in detail. While the high MI incidence risks of mild and severe MI among men and women with low incomes increased the expected number of life years after MI, this effect was counterbalanced by the comparably higher level of mortality after MI in the low-income group. This may also explain why the number of life years after severe MI did not differ by income group. For higher- income men, this even leads to spending more life years after mild MI than low-income men. In contrast, inequalities in incidence rates and mortality rates without MI have the same effect on MI-free life years: Both, increased incidence and increased mortality rates without MI, reduce MI-free life years in the low-income group.

Our results are consistent with previous studies that found substantial social inequalities in MI incidence and mortality risk [13–19, 31–34]. Moreover, our findings on income inequalities in MI severity support earlier findings reporting more severe MI events in individuals with low SES [17, 19, 21, 40–44]. Compared to the magnitude of inequalities in incidence and mortality without and after mild MI, the inequalities in death after severe MI were weaker, but still evident. This holds especially true for men. Given the high case fatality after a severe MI, this was to be expected. Sharing risk factors and similar pathogenetic processes, substantial income inequalities in life years free of stroke were also found for Germany in elderly women, and even more pronounced in elderly men [53].

Some studies suggest that the extent of inequalities in cardiovascular risk factors and harmful health behaviour may differ between genders, with inequalities being more pronounced in women than in men [24, 36]. For Germany, research on gender differences in health inequalities in risk factors and health behaviour is still limited but there is some evidence that inequalities in diabetes prevalence tend to be stronger in women than in men [36]. This contrasts with our findings which show greater inequalities in MI incidence and mortality among men than among women and underlines the importance of further research on how inequalities in risk factors for cardiovascular diseases affect inequalities in myocardial infarction incidence.

While inequalities in cardiovascular risk factors and health-related behaviour are often cited as most important factors in social inequalities in MI incidence [20, 23, 24], research is still needed on the underlying factors causing social inequalities in mortality after MI. In this context, there is some evidence that SES is an important predictor of how people can cope with the MI and its consequences, and of how they are able to adapt to the disease. In the context of a healthcare system granting nearly equal access to healthcare services for all individuals, the reasons for these mortality inequalities may lie in disparities in the adherence to long-term secondary and tertiary prevention and to changes in life style (e.g. [13, 32, 40]. Since income determines the financial resources available for daily living [49], it can be assumed that life style factors play an important role and foster inequalities in death after MI. Due to the relatively low mortality after mild MI found in our study (Additional file 1, Fig. S2), the probability of dying from causes other than MI can be assumed to be quite high compared to mortality after severe MI. This assumption may also be supported by the fact that the magnitude of inequalities between the income groups in mortality after mild MI lies between that of death without MI and that after severe MI. However, it remains unclear why inequalities in mortality after severe MI are higher in women than in men. These uncertainties indicate that further research is needed on the underlying mechanisms that cause these differences in mortality after acute myocardial infarction.

### Strengths and limitations

Our study is based on high case numbers of MI incidence and deaths without and after MI, which allow to conduct separate analyses for each of the five transitions and to stratify for different income groups. In addition, the detailed information on diagnosis permitted to examine income inequalities in MI incidence and mortality risks with respect to MI severity and thus to assess the length of life spent with less severe health impairments after mild MI and with more severe long-term impairments after severe MI due to chronic heart failure. Moreover, the statutory health insurance data used in this study include information on the precise date of diagnosis and death, which allows the exact determination of the time of exposure between the events required to perform multistate models. Furthermore, the data include individuals irrespective of their current health status. Therefore, our results are unaffected by health-related non-response which may occur in survey data when individuals refrain from study participation due to impaired health. Furthermore, there is no indication that diagnostic criteria differ between individuals with respect to income group. Since health insurance coverage is part of the well-fare state system and all costs are covered free of additional payments, it can be assumed that all individuals with MI are treated equally, that diagnostic methods do not differ between individuals with low and high income and that all income groups equally benefitted from the improvements in diagnostics over time.

Previous analyses have shown that the data are comparable to the total German population in terms of sex and age distribution but differ with respect to socioeconomic characteristics since individuals with high incomes and high occupational positions are underrepresented [47]. This was taken into account as all analyses are controlled for or stratified by income group.

Another advantage lies in the detailed information on individual income available in the data, which makes it possible to examine the gap in health expectancies between different income groups. Overall, income inequalities in MI incidence and mortality tend to be higher in men than in women. This finding is in line with results reported in other studies that analysed social inequalities in morbidity and mortality in the German population [31, 50, 52, 53]. However, some of the differences in the magnitude of inequalities may be attributed to the income measure used in this study. Since the data contain no information on household income, the available income may have been underestimated in some groups. Given the lower overall income level, this may apply particularly to women and might have led to an underestimation of health inequalities among females. However, comparative studies have shown that individual income is an appropriate SES indicator to measure health inequalities [54].

In our study, the level of physical impairments caused by the MI is depicted by including information on chronic heart failure. Individuals with a severe heart failure are assumed to be much more affected in their daily activities than those without or with a less severe heart failure. However, since the data do not contain any information about self-perceived impairments or health-related quality of life, the subjective assessment of the consequences of an MI could not be considered in our study. Previous research has shown that mortality after MI differs substantially by ST-segment elevation (STEMI) MI and non-STEMI (NSTEMI) MI [55]. However, these differences in mortality could not be analysed since the ICD-10 code do not allow for a clear differentiation between STEMI and NSTEMI MI.

## Conclusions

The study shows how income inequalities in MI incidence and mortality result in gaps in the length of life unaffected and affected by the consequences of MI. We found that income inequalities were most pronounced in MI-free life years. Due to greater inequalities in MI incidence and mortality, the gap in life years free of MI between income groups is considerably wider among men than among women. Furthermore, our findings suggest that inequalities in the years of life after MI, which are assumed to be spent in impaired health, are limited. Marked inequalities in life years spent after MI were only found for men after mild MI with a higher number of years for men with high incomes while no differences were found in life years after severe MI where impairments are likely to be strongest. Our findings underline the importance of public health efforts aimed at reducing health inequalities in cardiovascular diseases and related risk factors, promoting healthier lifestyles and strengthening secondary and tertiary prevention, with particular emphasis on men and women with high-risk profiles for MI incidence and cardiovascular mortality the most disadvantaged groups. Further research is needed to reveal the underlying factors explaining the SES differences in MI incidence and mortality with respect to MI severity.

## Supplementary Information

**Additional file 1.**

## Data Availability

The datasets generated and analysed during the current study are not publicly available due to protection of data privacy of the insured individuals by the Statutory Local Health Insurance of Lower Saxony (AOK Niedersachsen).

## Abbreviations

MI: Acute myocardial infarction
SES: Socioeconomic status
AOKN: AOK Niedersachsen
ICD-10: International Classification of Disease 10th revision
NYHA: New York Heart Association
STEMI: ST-segment elevation
