# Supplementary material for: Social inequalities in mild and severe myocardial infarction: how large is the gap in health expectancies?
Source: BMC Public Health. 2021 Feb 1;21:259. doi: 10.1186/s12889-021-10236-7 (PMC7852180; doi:10.1186/s12889-021-10236-7)
Supplement: Supplementary file 1 — Additional file 1. [file 12889_2021_10236_MOESM1_ESM.pdf]

# Additional file 1

## Social inequalities in mild and severe myocardial infarction: How large is the gap in health expectancies?

Figure S1 Income inequalities in the risks (HR) of myocardial infarction incidence and death by degree of myocardial infarction severity and gender (reference: higher-income group)

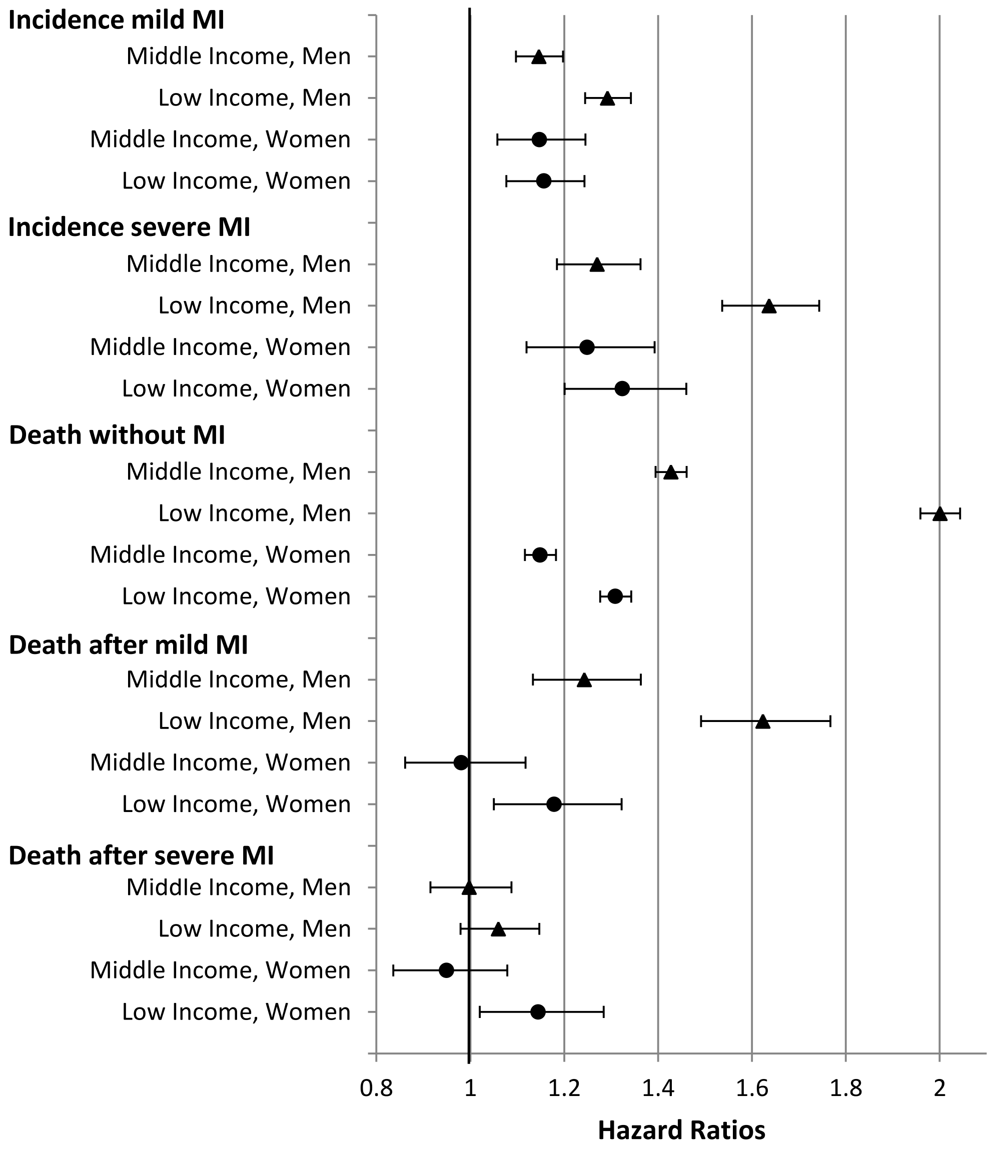


Note: HR Hazard Ratio; 95%-CI bootstrapped (with replacement) using 1000 replications; all analyses are controlled for age in single-year age groups (as second-degree polynomial)

Figure S2 Observed and predicted rates of myocardial infarction incidence by severity and income group

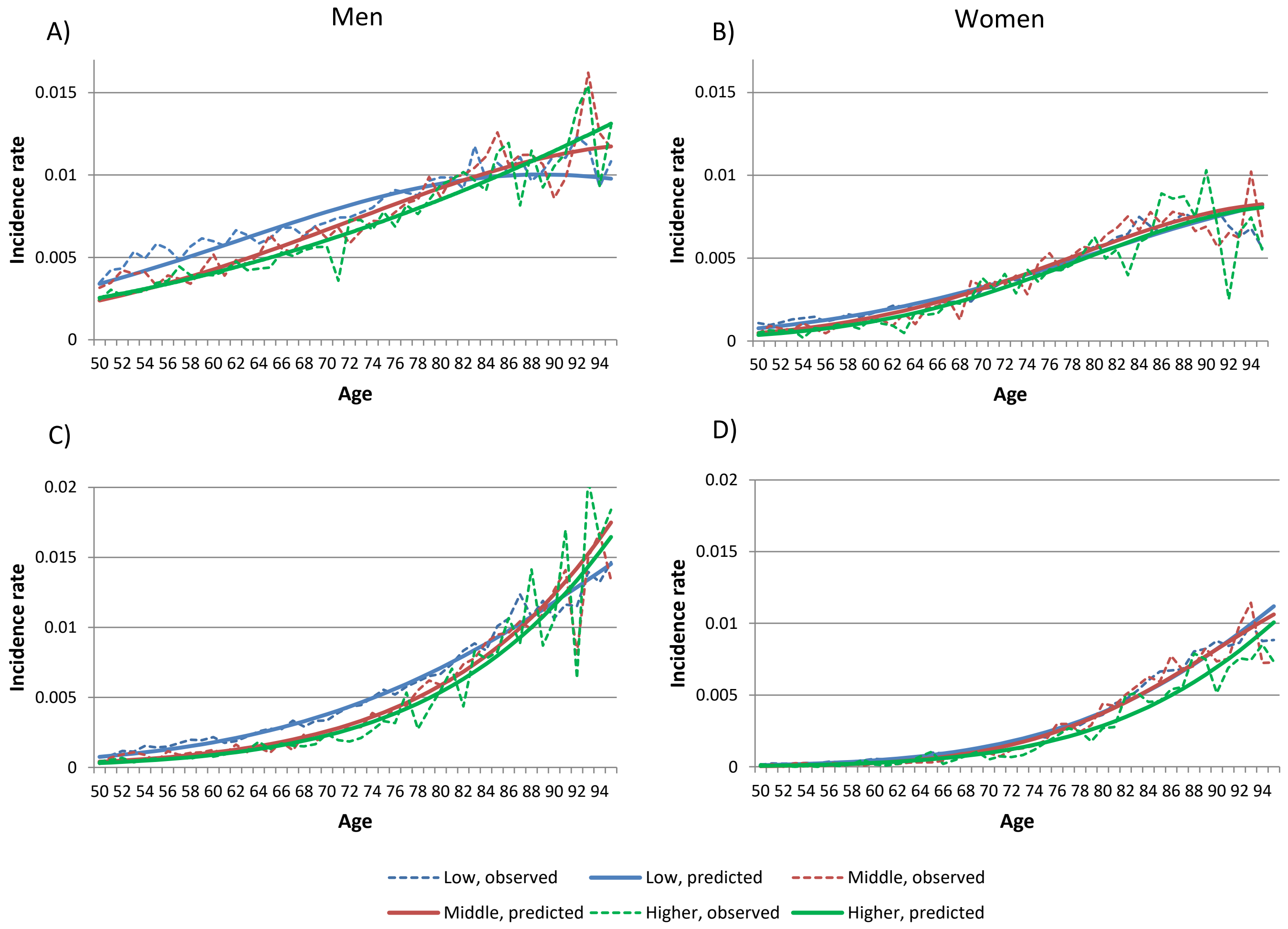


A) Incidence rate of mild myocardial infarction, men; B) incidence rate of mild myocardial infarction, women; C) incidence rate of severe myocardial infarction, men; D) incidence rate of severe myocardial infarction, women

Figure S3 Observed and predicted rates of death without MI, after mild MI, and after severe MI by income group

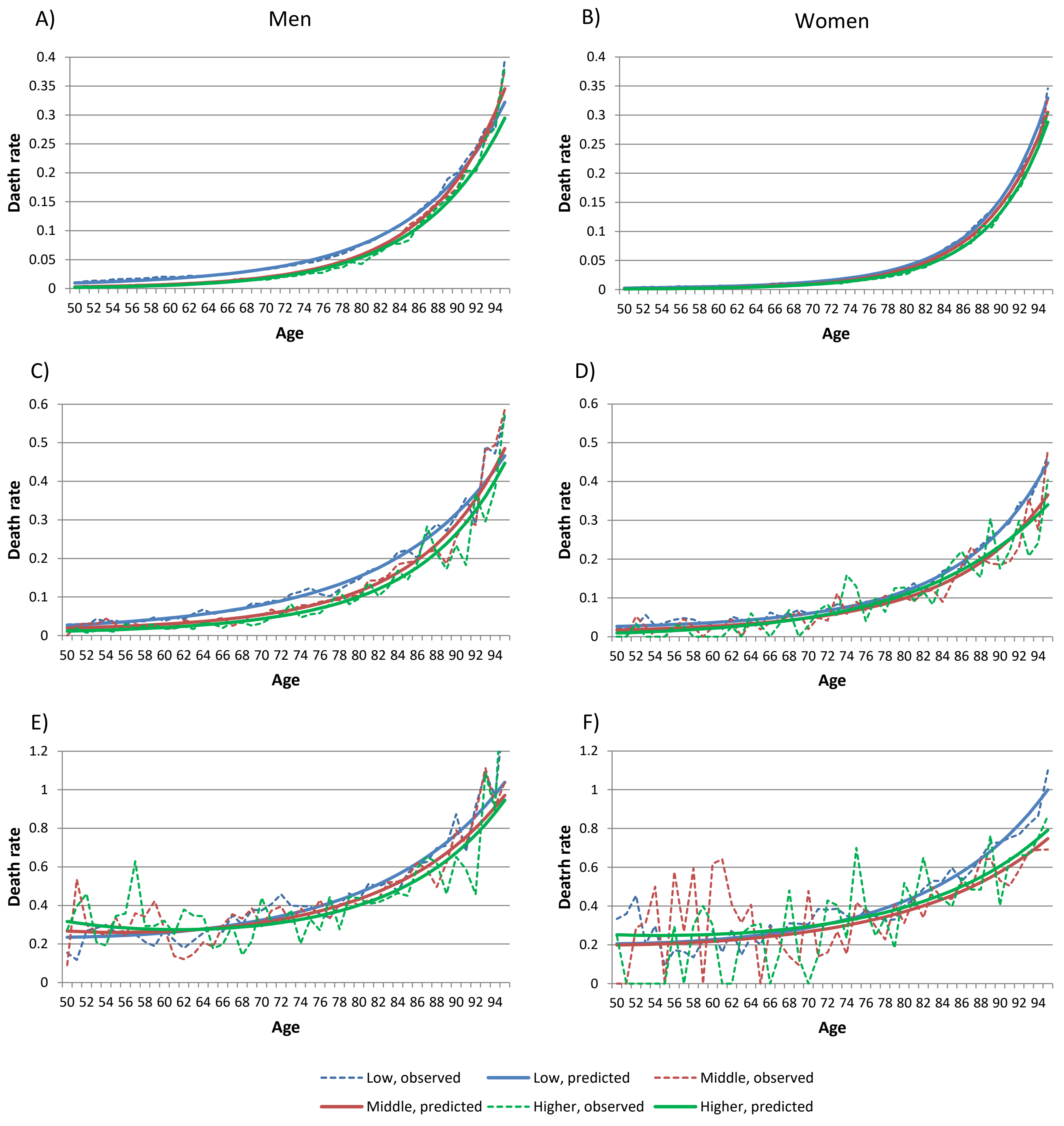


A) Death rate without myocardial infarction, men; B) death rate without myocardial infarction, women; C) death rate after mild myocardial infarction, men; D) death rate after mild myocardial infarction, women; E) death rate after severe myocardial infarction, men; F) death rate after severe myocardial infarction, women
